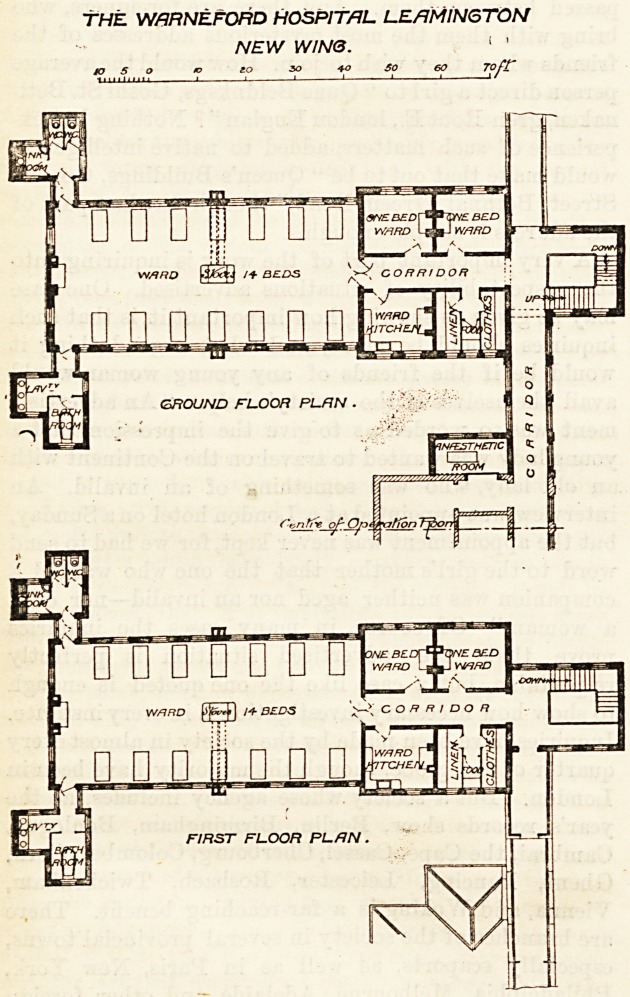# Hospital Construction

**Published:** 1900-08-25

**Authors:** 


### THE INSTITUTIONAL WORKSHOP.

### HOSPITAL CONSTRUCTION.

### THE NEW WING AT THE WARNEFORD HOSPITAL, LEAMINGTON.

This addition to the hospital is a two-storey block. Each floor consists of an oblong ward about 60 feet long and 26 feet wide. It is intended for 14 beds, and each bed has a window on both sides of it. Windows are also placed in the end walls, so that the ward is splendidly lighted, and cross-ventilation is easily carried out. The bath-room, closets, and sinks are placed in small blocks at the free ends of the wards, and they are correctly cut off from the mains by cross-ventilated passages. Attached to the ward are two single-bedded rooms, a ward kitchen and store-room for food, linen, and clothing. Excepting that the doors are placed nearly opposite the windows there is no proper cross-ventilation of the single-bedded rooms; but this is always a difficult, often an insoluble, problem in hospital architecture. Here, if the doors have fanlights made to open, the objection would not be very serious. The new wing is connected with the main building by a one-storey corridor, and part of it is so constructed that it can be used by the patients as a sun-room. It is much to be regretted that our infirmary corridors are not more frequently utilised for such purposes. An anæsthetic-room has been provided adjoining the operation-room at the point where the latter touches the new corridor. The wall of the staircase is so arranged that a lift can at any time be placed in it. Each ward has a stove in the centre, and an open fireplace at the end. This is the correct principle. The floors are of teak. The architects were Messrs. Young and Hall, of Southampton Street, Bloomsbury; and the contractors were Messrs. Smith and Sons, of Milverton, Leamington. The cost is not stated.

## Figures and Tables

**Figure f1:**